# Clinical manifestations of Long-COVID: an observational perspective

**DOI:** 10.3389/fmed.2025.1523817

**Published:** 2025-03-19

**Authors:** Muhammad Omair Sultan Meo, Noara AlHusseini, Dania Imad Ibrahim, Muhammad Zain Sultan Meo, Faridul Ahsan, Hani Tamim, Muhammad Raihan Sajid

**Affiliations:** College of Medicine, Alfaisal University, Riyadh, Saudi Arabia

**Keywords:** COVID-19, Long-COVID, post-COVID-19-syndrome, infectious disease, Saudi Arabia

## Abstract

**Objectives:**

Coronavirus Disease-19, also known as COVID-19, resulted in a pandemic that caused massive health concerns and economic losses globally. Worldwide, people are still facing persistent clinical symptoms following COVID-19 infection, characterized as Long-COVID. This study aimed to assess the experience and awareness of Long-COVID clinical symptoms in Saudi Arabia.

**Methods:**

This cross-sectional study was conducted at the College of Medicine, Alfaisal University, Riyadh, Saudi Arabia during the period April 5, 2023 to August 30, 2023. An online questionnaire was created using Google Forms and distributed with a URL to students via email and WhatsApp. The questionnaire consisted of 17 questions classifying the respondent’s responses. The survey consisted of three sections, addressing demographics, their experience with COVID-19 and whether they had Long-COVID symptoms. A total of 490 participants participated in the study survey. The Statistical Package for Social Sciences (SPSS) version 28 was used for data administration and analysis. *P*-values <0.05 were considered statistically significant.

**Results:**

Out of the participants, 53.9% had prior exposure to COVID-19. During acute infection, tiredness was the most common symptom in participants, observed in 84.5% of people. The other common symptoms which were reported among the participants included fever (75%), soreness of throat (65.5%), headache (65.2). Some symptoms were more dominant in males (such as acne) and some in females (hair loss). Regarding prolonged symptoms, 43.6% of females and 33.3% of males had experienced symptoms of Long-COVID. Tiredness was once again the most dominant symptom (16.7%). The other common prolonged symptoms of Long-COVID observed were loss of taste or smell (9.1%), body pain (7.6%), headache (7.2%), foggy memory (7.2%) and shortness of breath (6.4%). Lastly, awareness of Long COVID was lower than expected, with 51.5% of females and 55.1% of males unaware of the syndrome.

**Conclusion:**

This study’s findings show the effects Long COVID-19 has on the general population, which includes various symptoms affecting physical, mental and emotional needs. The findings also suggest potential gender differences in Long-COVID clinical symptoms, thus highlighting the need for further research with larger and more diverse samples.

## Introduction

Coronavirus Disease-19, also called COVID-19, resulted in a global pandemic, disrupting healthcare systems and affecting millions worldwide (1, 2). While the initial illness is often characterized by respiratory symptoms, growing evidence suggests that some individuals experience lingering effects beyond the acute phase. This condition, known as post-COVID syndrome or Long-COVID, can significantly impact various organ systems. Long COVID-19, also known as “post-acute sequelae of COVID-19,” is a multisystemic illness that develops after a severe acute respiratory syndrome coronavirus 2 (SARS-CoV-2) infection. This condition is characterized by multiple severe symptoms. Based on a conservative projected incidence of 10% of infected persons with more than 770 million recorded COVID-19 cases globally (as per World Health Organization’s data); at least 77 million people worldwide have Long COVID; the figure could be much higher due to numerous unregistered instances. 10%–30% of non-hospitalized cases, 50%–70% of hospitalized patients, and 10%–12% of vaccinated cases are thought to have the incidence (3, 4).

Most Long COVID cases are in non-hospitalized patients with mild acute illness, as this population represents most overall COVID-19 cases. Long COVID is associated with all ages and acute phase disease severities, with the highest percentage of diagnoses between the ages of 36 and 50 years (3). One of the most commonly used timeframes for Long COVID is symptoms present 4 weeks after the initial acute infection, as used by the CDC (Centers for Disease Control and Prevention) (5, 6). Literature indicates that Long-COVID can manifest in various physical and neuropsychiatric symptoms lasting much longer after recovery from the initial infection. The severity of the initial COVID-19 illness does not necessarily predict the likelihood of developing Long-COVID (7). The most frequently reported symptoms include fatigue, muscle weakness, headaches, joint pains, chest pain, cognitive decline and memory loss, and other neuropsychiatric disorders like anxiety, sleep disorders (3, 7–10). ICU admission in patients with COVID-19 further increases the risk of developing psychological issues like anxiety, depression, headache and post-traumatic stress disorder (PTSD) (11, 12).

Moreover, there could be various factors that cause differences between symptoms experienced during Long-COVID. One such factor could be gender related. Many studies have suggested that the prevalence of Long-COVID tends to be higher among females. Some suggest that certain hormones in females may be the cause behind the hyperinflammatory status of the acute COVID phase, which then lasts long after recovery, resulting in these symptoms (13).

While the exact cause of Long-COVID remains unclear, factors like age, underlying health conditions (comorbidities), and frailty might influence susceptibility (11, 14). Studies have shown that patients with pre-existing or chronic conditions, such as diabetes, chronic lung diseases, asthma, chronic kidney disease, would be at a higher risk of developing this syndrome (15).

Given the evolving nature of Long-COVID research, a comprehensive understanding of its prevalence and specific manifestations is crucial. A literature search showed a paucity of studies from Saudi Arabia assessing the frequency of this syndrome in this country. Therefore, this study aims to assess the presence of post-COVID symptoms among individuals who have previously contracted COVID-19 in Saudi Arabia and to investigate any potential gender differences.

## Materials and methods

For this cross-sectional study, we created an online questionnaire using Google Forms and distributed a URL to students via email and WhatsApp from April 5, 2023, until August 30, 2023. The targeted audience for this study was people who were diagnosed with COVID-19 (aged 18 years or older) living in Saudi Arabia.

To achieve the study’s goals, the developed questionnaire was modified from the paper “Characteristics and impact of Long Covid: Findings from an online survey” (16). Moreover, the questionnaire survey was submitted to 6 senior faculty members, and they filled the survey to identify any technical issues. Hence it enhanced the validity of the questionnaire, since the faculty reviewed its contents and ensured the survey was clear and concise. It was aimed at Saudi Arabian citizens and residents who were diagnosed with COVID-19, and all participants who did not meet those requirements were excluded from the study. With 32.2 million people living in Saudi Arabia, we estimated that our target sample should consist of 385–400 people to attain a 95% confidence level with a 5% margin of error using the standard formula for sample size calculation via the Raosoft calculator tool (17).

The questionnaire consisted of 17 questions classifying the respondent’s responses. We asked participants to answer questions regarding the symptoms they experienced when infected with COVID-19 and the long-term symptoms that stayed with them. The survey consisted of three sections. The first section addressed demographic aspects, including gender, age, current education level, monthly income, employment status, nationality, region they reside in, and whether they caught COVID-19 previously. The second section comprised questions querying participants about the frequency of their infections, their experience of symptoms, and the specific symptoms encountered. The third section included questions about their awareness of post-COVID-19 and other symptoms they encountered.

### Statistical analysis

We retrieved data from the survey and manually reviewed it to ensure it met the inclusion requirements. We methodically arranged the data using Microsoft Excel into groupings of symptoms, demographic characteristics, and independent variables. Whereas continuous variables were shown as mean and standard deviation, categorical data were expressed as numbers and percentages. Chi-square tests were used to examine relationships between various parameters. This test was selected because our dataset mainly comprises of categorical variables (e.g., age groups, education level, nationality), which made Chi-squares appropriate for investigating potential associations between categorical independent and dependent variables. The Statistical Package for Social Sciences (SPSS) version 28 was used for data administration and analysis. *P*-values < 0.05 were considered statistically significant.

### Ethical approval

This study has received ethical approval from Alfaisal University’s Institutional Review Board (IRB-20209). Participation was anonymous, discreet, and entirely optional. Informed consent was obtained from all participants. To maintain anonymity, no names or other identifiable information was gathered.

## Results

This study included a total of 490 participants, categorized by age, education level, social status, employment, income and nationality (Table 1). Out of these participants, 53.9% had prior exposure to COVID-19 (Table 2). Looking into genders, 195 females (55.1%) and 69 males (50.7%) had prior COVID-19 exposure, showing that over half of the participants had prior exposure. Amongst these participants, 16.9% of men and 15% of women had been exposed to COVID-19 at least twice. Moreover, 43.6% of females had experienced prolonged symptoms, along with 33.3% of males. And lastly, awareness of Long COVID was lower than anticipated, with 51.5% of females and 55.1% of males unaware of the condition.

**TABLE 1:** Demographics of the survey participants.

	Total participants	Female (*n*)	Male (*n*)	*p*-value
**Age**				
18–29	356 (72.7)	248 (70.1)	108 (79.4)	0.008
30–44	101 (20.6)	85 (24.0)	16 (11.8)	
45+	33 (6.7)	21 (5.9)	12 (8.8)	
**Education level**				
High school (or below)	123 (25.1)	89 (25.1)	34 (25.0)	0.857
Undergraduate	318 (64.9)	228 (64.4)	90 (66.2)	
Postgraduate	49 (10.0)	37 (10.5)	12 (8.8)	
**Social Status**				
Single	375 (76.5)	260 (73.4)	115 (84.6)	0.006
Divorced	1 (0.2)	0 (0.0)	1 (0.7)	
Married	114 (23.3)	94 (26.6)	20 (14.7)	
**Employment**				
Employed	93 (19.0)	66 (18.6)	27 (19.9)	0.007
Student	339 (69.2)	236 (66.7)	103 (75.7)	
Unemployed	58 (11.8)	52 (14.7)	6 (4.4)	
**Monthly income**				
9,999 or less	86 (17.6)	57 (16.1)	29 (21.3)	0.152
10,000–20,000	41 (8.4)	32 (9.0)	9 (2.5)	
20,000 +	31 (6.3)	19 (5.4)	12 (8.8)	
Prefer not to say	101 (20.6)	77 (21.8)	24 (17.6)	
Do not have an income	231 (47.1)	169 (47.7)	62 (45.6)	
**Nationality**				
Non-Saudi	317 (64.7)	217 (61.3)	100 (73.5)	0.011
Saudi	173 (35.3)	137 (38.7)	36 (26.5)	

**TABLE 2:** The participants who suffered from COVID-19 previously, and whether they were aware of Long COVID and clinical symptoms.

	Total participants	Female (*n*)	Male (*n*)	*p*-value
**Prior exposure to COVID-19**				
Yes	264 (53.9)	195 (55.1)	69 (50.7)	0.387
No	226 (46.1)	159 (44.9)	67 (49.3)	
**Frequency of exposure**				
0	226 (46.1)	159 (44.9)	67 (49.3)	0.437
1	188 (38.4)	142 (40.1)	46 (33.8)	
2+	76 (15.5)	53 (15.0)	23 (16.9)	
**Awareness of Long COVID**				
Yes	125 (47.5)	94 (48.5)	31 (44.9)	0.614
No	138 (52.5)	100 (51.5)	38 (55.1)	
**Experienced prolonged symptoms**				
Yes	108 (40.1)	85 (43.6)	23 (33.3)	0.136
No	156 (59.1)	110 (56.4)	46 (66.7)	

During acute infection, tiredness was the most common symptom in participants, reported in 84.5% of people. The other common symptoms among the participants included fever (75%), soreness of throat (65.5%), headache (65.2%), body aches (63.6%), cough (55.3%), loss of taste/smell (48.1%) (Table 3). Participants also reported various symptoms related to mental health during the infection, with fatigue reported by 1/3rd of participants. Others included anxiety (21.2%), stress (18.9%), issues with sleep (18.9%), lack of focus (10.2%), and finally PTSD (1.9%). It was noted that some symptoms were more commonly reported by one gender over the other. Acne was reported in 15.9% of males versus 4.1% of females, a significant difference (*p* = 0.001). This could possibly be due to an underlying mechanism of biological and hormonal factors, as males typically have higher levels of androgens which are known to contribute to acne development. Further research needs to be conducted to further understand the biological mechanism behind this effect and its association with Long Covid. Furthermore, hair loss was reported more often in females (23.6%) than males (8.7%), a difference of 14.9% between the two (*p* = 0.008). This finding supports another study, which showed females (82.8%) having predominant hair loss after diagnosis of COVID-19 in comparison to males (17.2%) (18). Female sex hormones like progesterone and estrogen could have a role in underlying pathophysiology, and COVID-19 may cause a reduction in these systemic hormones in female patients (18). Future studies are needed to investigate a relationship between the extent of hair loss and female sex hormones.

**TABLE 3:** Clinical symptoms faced by COVID-19 patients during their acute infection period.

Characteristics	Symptoms in all participants	Symptoms in females (*n*)	Symptoms in males (*n*)	*p*-value
Tiredness	223 (84.5)	167 (85.6)	56 (81.2)	0.377
Fever	198 (75.0)	145 (74.4)	53 (76.8)	0.686
Soreness of throat	173 (65.5)	128 (65.6)	45 (65.2)	0.949
Headache	172 (65.2)	129 (66.2)	43 (62.3)	0.566
Body ache/pain	168 (63.6)	130 (67.0)	38 (55.1)	0.076
Cough (dry)	146 (55.3)	102 (52.3)	44 (63.8)	0.100
Loss of taste/smell	127 (48.1)	87 (44.6)	40 (58.0)	0.056
Cough (with sputum)	78 (29.5)	60 (30.8)	18 (26.1)	0.464
Shortness of breath	78 (29.5)	58 (29.7)	20 (29.0)	0.906
Runny nose	56 (21.2)	42 (21.5)	14 (20.3)	0.827
Hair loss	52 (19.7)	46 (23.6)	6 (8.7)	**0.008**
Nausea	43 (16.3)	30 (15.4)	13 (18.8)	0.504
Foggy memory	28 (10.6)	22 (11.3)	6 (8.7)	0.549
Diarrhea	20 (7.6)	12 (6.2)	8 (11.6)	0.142
Acne	19 (7.2)	8 (4.1)	11 (15.9)	**0.001**

When it came to symptoms faced after recovery from COVID-19, tiredness was once again the most dominant symptom among the participants (16.7%) (Table 4). The other common symptoms were loss of taste or smell (9.1%), body pain (7.6%), headache (7.2%), foggy memory (7.2%) and shortness of breath (6.4%).

**TABLE 4:** Clinical symptoms of Long-COVID faced after COVID recovery from the patients who had prior infection.

Characteristics	Symptoms in all participants	Symptoms in females (*n*)	Symptoms in males (*n*)	*p*-value
Tiredness	44 (16.7)	33 (16.9)	11 (15.9)	0.851
Loss of taste/smell	24 (9.1)	14 (7.2)	10 (14.5)	0.069
Body ache/pain	20 (7.6)	16 (8.2)	4 (5.8)	0.516
Headache	19 (7.2)	15 (7.7)	4 (5.8)	0.601
Foggy memory	19 (7.2)	14 (7.2)	5 (7.2)	0.985
Shortness of breath	17 (6.4)	15 (7.7)	2 (2.9)	0.163
Cough (dry)	15 (5.7)	9 (4.6)	6 (8.7)	0.208
Hair loss	9 (3.4)	7 (3.6)	2 (2.9)	0.786
Cough (with sputum)	7 (2.7)	5 (2.6)	2 (2.9)	0.882
Acne	6 (2.3)	2 (1.0)	4 (5.8)	0.022
Runny nose	3 (1.1)	3 (1.5)	0 (0.0)	0.300
Fever	2 (0.8)	1 (0.5)	1 (1.4)	0.441
Nausea	2 (0.8)	2 (1.0)	0 (0.0)	0.398

Regarding mental-health related symptoms faced after recovery from infection, our study showed that almost 21.2% of participants faced anxiety/depression, and 18.9% of participants reported stress along with sleep problems (Table 5). This underscores the prolonged impact COVID has on mental health.

**TABLE 5:** Mental health-related symptoms faced after COVID recovery (Prolonged).

Symptoms	Symptoms in all participants	Females (*n*)	Male (*n*)	*p*-value
Fatigue	87 (33.0)	65 (33.3)	22 (31.9)	0.826
Anxiety/depression	56 (21.2)	43 (22.1)	13 (18.8)	0.575
Stress	50 (18.9)	38 (19.5)	12 (17.4)	0.703
Sleep problems	50 (18.9)	35 (17.9)	15 (21.7)	0.490
Lack of focus/concentration	27 (10.2)	20 (10.3)	7 (10.1)	0.979
Post-traumatic stress disorder	5 (1.9)	4 (2.1)	1 (1.4)	0.753

## Discussion

This study aimed to investigate the experience of Long-COVID symptoms among individuals who previously contracted COVID-19 in Saudi Arabia. The analysis revealed some interesting trends regarding demographics. There was a statistically significant difference in the age distribution (*p* = 0.008), with females more likely to be in the 18–29 age group compared to males. Social status showed a significant difference (*p* = 0.006), with a higher proportion of single individuals among females compared to males. Additionally, employment status differed significantly (*p* = 0.007), with more females being students and fewer being employed compared to males. Nationality also differed significantly (*p* = 0.011), with a higher proportion of non-Saudi participants being female.

### Symptoms during acute COVID-19 infection

In literature, various studies have highlighted the differences COVID has on the symptomatology between genders. A study in 2023 showed females having significantly higher mean scores for headache, nasal congestion, and appetite problems (19). Another study in Arkansas (20) showed that males were more likely to report experiencing fever and chills compared to females at the time of testing.

Examining our results, it was interesting to note that some symptoms were more commonly reported by one gender over the other. During the acute infection, males were more likely to report dry cough (63.8%) compared to 52.3% of females, as well as loss of taste/smell (58.0% of males and 44.6% of females, *p* = 0.056). Acne was also observed in 15.9% of males versus 4.1% of females, a significant difference (*p* = 0.001). Furthermore, hair loss was observed more often in females (23.6%) than males (8.7%), a difference of 14.9% between the two (*p* = 0.008). Overall, there were significant gender differences for a few symptoms experienced during the initial COVID-19 infection.

### Symptoms of long COVID-19

Most reported symptoms of Long Covid were tiredness or fatigue, loss of taste and/or loss of smell, body ache/pain, headaches, shortness of breath, prolonged dry cough etc. These findings are similar to previously published studies from the Gulf and other countries (8, 10, 16). Prolonged fatigue has been commonly reported as a significant symptom of long COVID-19 in multiple studies previously (21–23). The possible mechanisms underlying fatigue include inflammation, sleep alterations, autonomic nervous system abnormalities, and poor nutritional status, all of which are also a common thread of COVID-19 infection (24). In a similar study, the majority of patients exhibited a strong relapsing aspect of these symptoms daily (16). Numerous participants named stress, physical or mental exertion, sleep disturbance, and household tasks as triggers for their symptoms. Changing daily routines to accommodate the avoidance of symptom-triggering activities is necessary. Some individuals may manage these adjustments because of their jobs and personal situations, while others may struggle, making them feel worse. These findings highlight the burden of Long COVID on the patients, and further emphasize investigated for remedial measures (16).

In a recent review looking at long-term health, 63% of the cohort reported feeling tired or weak in their muscles, 26% reported having trouble sleeping, and 23% reported having anxiety or despair. More severe COVID-19 infections were associated with neurological symptoms such as skeletal muscle damage, acute cerebrovascular disorders, and reduced awareness (11). Another Long-COVID study which included respondents from 56 nations, the majority of whom were Americans, revealed that Long-COVID impacts several organ systems, with exhaustion and cognitive impairment being the most frequently reported long-term symptoms (21). Physical activity, mental activity, and stress were prominent triggers for the recurrence or aggravation of symptoms; however, the survey also revealed sleep deprivation as a prevalent cause (16, 21).

It’s interesting to note that after the Middle East Respiratory Syndrome (MERS) outbreak in 2012, as well as the SARS-CoV-1 outbreak in 2003, comparable observations of long-term consequences have been documented (25, 26). A type of post-acute viral syndrome was observed in the survivors of the first two CoV outbreaks, who reported chronic dyspnea, exhaustion, mental health issues, and a decreased quality of life (25, 26).

Looking at prolonged symptoms in our study, a higher number of females (43.6%) faced Long COVID symptoms in comparison to males (33.3%) [but this difference wasn’t statistically significant (*p* = 0.136)]. However, many studies in literature report that females are more likely to develop Long-COVID. Some hypothesize that certain physiologic hormones in females would make the acute phase of the disease to last longer than normal. Conversely, one study found that women tend to develop higher levels of immunoglobulin G (IgG) during infection, which could help develop a more favorable outcome (15). However, certain demographic factors may also lead to this. Women tend to follow-up more with their care, and exhibit greater awareness of their health (13).

**FIGURE 1:**
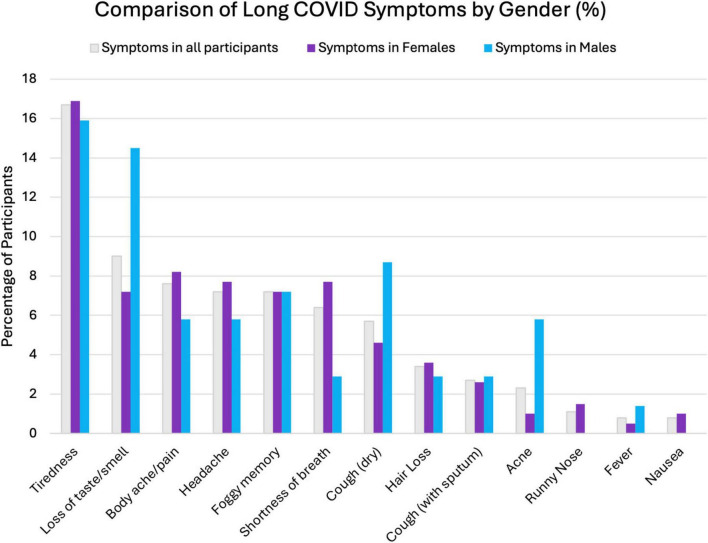
Comparing Long COVID symptoms between all participants, males, and females.

In our study, some symptoms were more commonly reported by females, while others were more dominant in males. Body pain was reported by 8.2% of females in contrast with 5.8% of males. However, there was a notable difference between genders for loss of taste once again, with 14.5% of males facing the symptom compared to 7.2% of females (*p* = 0.069). A significant finding was found for acne, with 5.8% of males facing it compared to only 1% of females. This data has been visualized in our graph (Figure 1) above. These findings suggest the effects long COVID-19 might have on different genders, and further studies are needed to explore this dynamic.

### Psychological impairments

Many of the respondents reported neurological and psychological symptoms like sleep deprivation, inability to concentrate, and mental health problems like delirium, brain fog, memory loss, hallucinations, confusion, melancholy, and anxiety; similar findings have been reported in other studies (27, 28). Almost 1/5th of the participants in our study reported feeling anxiety/depression after recovery, which demonstrates the negative consequences Long COVID may have in the future on mental health. These results also match those of a meta-analysis that showed a high prevalence of depression, anxiety, and insomnia in patients recovering from COVID-19 (29). A meta-analysis encompassing 12 research articles and 4828 cases of post-acute COVID-19 syndrome (PCS) revealed a correlation between low quality of life, persisting symptoms, and mental health (30).

### Awareness of Long COVID-19

When it came to awareness about Long COVID-19, an astounding 47.5% of people were unaware of this issue. According to another survey that was conducted, there was limited awareness of Long COVID and marked disparities in knowledge when it came to ethnicities and language. This poses the risk of delayed diagnosis and treatment and could underestimate the long-term impact of Long COVID-19 on the population. Reasons behind this lack of awareness could include various socioeconomic differences between regions. As an example, in the US, Long COVID awareness was lower in populations who had low proficiency in English (31). Another study showed that the awareness of Long-COVID was lower in certain races such as African Americans, and Latinos. Awareness was also lower among patients who did not have primary care (32).

### Study limitations

Similar to other studies, our study has a few limitations. The reliance on self-reported data can introduce recall bias. The online survey format may have limited participation from individuals without internet access, and older individuals, since younger people are more likely to engage in digital surveys. This could limit the generalizability of our findings. Moreover, the sample size for men is smaller than that of females since more females responded to the survey.

## Conclusion

Globally, more than 27 million people have recovered from COVID-19, but little is known about the disease’s long-term effects. According to preliminary research, some patients may have long-lasting anomalies in their physiological systems. Our results underscore the impact that COVID-19 has on patients long after they have been reported to be well from acute symptoms of the illness, along with the gender disparity in symptomatology experienced; they also show the necessity of a thorough, multidisciplinary approach to provide these patients with the best care possible. Nevertheless, larger and more diverse samples are needed to confirm these findings. Clinicians should be aware of the long-term effects of COVID-19, and the potential differences that may be observed between different genders. Studies investigating the biological mechanisms underlying these gender differences are warranted.

## Data Availability

The raw data supporting the conclusions of this article will be made available by the authors, without undue reservation.
